# Does the application of Jensen's integral inequality and LMIs confirm exponential stability in delayed systems with gapped gamma distribution through augmented Lyapunov function?

**DOI:** 10.1016/j.heliyon.2024.e39077

**Published:** 2024-10-09

**Authors:** Sara Mahmoudi Rashid

**Affiliations:** Faculty of Electrical and Computer Engineering, University of Tabriz, Tabriz, Iran

**Keywords:** Exponential stability, Delayed systems, Gapped gamma distribution, Jensen's integral inequality, Linear matrix inequalities (LMIs), Augmented lyapunov functions

## Abstract

This study is dedicated to a comprehensive exploration aimed at advancing our understanding of stability within dynamic systems. The focus is particularly on the intricate domain of delayed systems characterized by gapped gamma distributions. The primary objective of this investigation revolves around evaluating the pragmatic application and efficacy of Jensen's integral inequality in combination with the powerful analytical tools provided by Linear Matrix Inequalities (LMIs). This evaluation is crucial for rigorously assessing exponential stability within these complex systems. Central to our investigative framework is the strategic deployment of augmented Lyapunov functions. These functions play a crucial role in unraveling the intricate stability properties of delayed systems featuring gapped gamma distributions, allowing for a nuanced examination of their inherent stability characteristics under various conditions. The mathematical formulation crafted in this exploration intricately captures the interplay between the distinctive attributes of the gapped gamma distribution and the complex dynamics of the loop traffic flow model within the overarching delayed system. This interconnection serves as the fundamental basis for the stability analysis, providing insights into the interdependence of these key elements. The noteworthy contribution of this study lies in the systematic construction of a robust analytical framework explicitly tailored for stability assessment. A comprehensive investigation is undertaken to elucidate critical aspects, including the convergence rate and the attainment of asymptotic stability within the considered delayed system. Additionally, a dedicated simulation section, focusing on Vehicle Active Suspension Control, has been incorporated to further validate and showcase the applicability of the proposed methodology.

## 1 Introduction

### 1.1 Motivation

In the continually evolving realm of dynamical systems [1], the persistent pursuit of understanding stability intricacies stands as an unceasing endeavor [2]. Within this pursuit, this study ventures into the intricate domain of delayed systems distinguished by gapped gamma distributions. The impetus driving this exploration emanates from the pivotal significance of stability analysis that reverberates across diverse scientific spheres [3]. Spanning from engineering to biology, dynamic systems constitute the bedrock for comprehending real-world phenomena, underscoring the urgency of unraveling their stability dynamics [4].

The examination of delayed systems [5], characterized by temporal lags in their responses, has captured substantial scholarly attention due to their pervasive presence in a myriad of practical contexts [6]. These systems frequently showcase intricate behaviors that defy conventional stability analysis techniques [7]. Gapped gamma distributions, marked by their distinctive attributes and broad applicability across diverse domains, emerge as an alluring contender for capturing temporal interdependencies inherent in these systems. However, despite their potential, the comprehensive exploration of their stability properties remains a frontier awaiting meticulous scrutiny.

The primary motivation for this research is to enhance our understanding of the stability of delayed systems, particularly those characterized by gapped gamma distributions. Delayed systems are prevalent in numerous real-world applications, including engineering, economics, and biological systems. These systems often exhibit complex behaviors due to time delays, which can significantly affect their stability and performance.

One practical application that exemplifies the significance of this research is vehicle active suspension control. In such systems, delays can arise from sensor processing, actuator response, and communication lags. Ensuring the stability of the suspension system despite these delays is crucial for maintaining vehicle safety and passenger comfort.

By applying Jensen's Integral Inequality and LMIs, we aim to develop a robust analytical framework for confirming the exponential stability of delayed systems. The use of an Augmented Lyapunov Function in our approach allows for a more precise and comprehensive stability analysis, accommodating the unique challenges posed by gapped gamma distributions.

Our study's findings are particularly relevant to the automotive industry, where improving active suspension systems can lead to significant advancements in vehicle dynamics and safety. The simulation results, including the newly added section on vehicle active suspension control, demonstrate the practical applicability and effectiveness of our proposed method. This underscores the broader impact of our research on enhancing the reliability and performance of delayed systems across various domains.

**Table:** Nomenclatures

x(t)	The state vector of the system at time t
$\dot{x}\left(t\right)$	The time derivative of the state vector x(t) Denoting the rate of change of the system's state over time.
A	The system matrix, which governs the linear dynamics of the state x(t)
$A_{d}$	A matrix that influences the evolution of the state x(t) through the convolution integral with the delay kernel
Γ(θ)	The gamma distribution delay kernel
τ	The delay parameter, which introduces a time delay
t	Refers to the current time instant
θ	Symbolizes the integration variable within the convolution integral, spanning over the range of [0,∞)
N	The Size parameter of the gamma distribution
T	The scale parameter of the gamma distribution
$V_{aug}$	Augmented Lyapunov function
δ	The convergence rate

### 1.2 Literature review

Significant scholarly endeavors have been dedicated to the intricate domain of stability analysis within dynamical systems, spanning diverse academic disciplines including control theory, mathematics, and engineering [8]. This collective pursuit has yielded profound insights, forming the bedrock for comprehending the stability intricacies inherent in dynamic systems. Central to this domain, Lyapunov-based methodologies have been recurrently employed to establish fundamental stability conditions [9]. A contemporary advancement in the realm of stability analysis is the concept of augmented Lyapunov functions [10,11]. This innovation provides a potent toolset, enabling the exploration of intricate stability challenges within complex systems [12,13]. By their augmentation, Lyapunov functions attain heightened versatility, enabling a sophisticated dissection of system behaviors under intricate conditions [14,15]. Of note is the application of Jensen's integral inequality, a mathematical construct of substantial repute for bounding nonlinear terms [16,17]. This tool has effectively demonstrated its prowess in the realm of stability analysis [[18], [19], [20]]. Its application is grounded in its capacity to provide upper bounds to intricate non-linear functions, thus facilitating the derivation of stability criteria [21].

Time delays are commonly seen as a source of instability and reduced performance in dynamic systems. However, recent research suggests that under certain conditions, appropriate time delays can enhance system performance. For instance, the study [22], presents a comprehensive analysis of sampled-data systems with time-delay. By using a two-sided looped-functional approach, the authors derive improved conditions for system stability and reveal intrinsic relationships between sampled-data periods and time delay. Contrary to traditional views, their simulations demonstrate that time delays can enlarge the interval of sampled-data periods and accelerate the rate of convergence of the system states, rather than deteriorate system performance. These findings challenge the conventional perspective on time delays and highlight their potential benefits in dynamic systems.

Building on these insights, our study investigates the exponential stability of delayed systems characterized by gapped gamma distributions. By employing advanced analytical tools such as Jensen's Integral Inequality and LMIs, we aim to develop a robust framework for stability analysis. The inclusion of an Augmented Lyapunov Function further enhances our methodology, enabling a more detailed and accurate assessment of system stability.

Moreover, LMIs have surged to prominence as a versatile approach for substantiating stability conditions [23]. This methodology's versatility stems from its adaptability to accommodate various system dynamics, rendering it a preferred choice in diverse analytical contexts. Nonetheless, within the intricate domain of delayed systems featuring gapped gamma distributions, a realm where temporal dependencies intertwine with complex distributional characteristics, the harmonious integration of these methodologies remains relatively uncharted territory. The synergistic exploration of augmented Lyapunov functions, Jensen's integral inequality, and LMIs within such a context is a promising yet underexplored avenue. In light of these considerations, this study sets out to bridge this gap by holistically integrating these analytical tools within the complex framework of delayed systems characterized by gapped gamma distributions. This endeavor seeks to unearth the nuanced stability properties of such intricate systems and contribute to a deeper understanding of their behaviors under temporal dependencies and distributional variations. Through this exploration, the study aspires to extend the frontiers of stability analysis, paving the way for more comprehensive and robust analytical approaches within this intricate realm.

In reference [24], the method investigates the influence of Poisson-distributed delays on system stability, offering insights into their interaction with system behaviors in real-world scenarios. While advantageous for diverse applications, the approach's potential drawback is the complexity arising from the probabilistic nature of Poisson-distributed delays, demanding advanced mathematical analyses. Article [25] introduces innovative generalized Halanay inequalities for analyzing stability in nonlinear non-autonomous time-delay systems. These inequalities provide a powerful tool to address challenges arising from nonlinearity, time-delay, and non-autonomous dynamics, offering broad applicability across engineering, physics, and biology. The method enhances stability evaluation in complex systems, yet potential limitations include the mathematical complexity of deriving these inequalities, requiring advanced techniques for manipulation. A novel framework of generalized Jensen inequalities is introduced to analyze stability in article [26] in systems with distributed delays and infinite time-horizons. This method offers versatility across practical domains like control systems and economics, addressing complex scenarios with distributed delays. However, potential limitations may arise from increased mathematical complexity, particularly when dealing with infinite time-horizons.

Article [27] employs a Lyapunov-based approach to analyze the stability of systems with gamma-distributed delays, addressing diverse temporal dependencies. While the method captures complex delay patterns applicable to engineering and biology, challenges may arise from mathematical intricacy and computational requirements.

The Bessel–Laguerre inequality for stability analysis of systems with infinite distributed delays, offers insights beyond conventional methodologies introduced in article [28]. While versatile for control systems and communication networks, potential challenges include mathematical complexity in handling infinite delays. Article [29] introduces a refined Jensen-based inequality approach, enhancing the analysis of stability in time-delay systems by capturing the impact of various delay distributions. While applicable across fields, a challenge lies in formulating and solving these refined inequalities, potentially requiring advanced mathematical techniques. Stability analysis in distributed cyber-physical power systems, employing a meticulous modeling framework to assess small-signal stability with time-delay dynamics explores in the article [30]. While applicable in power systems engineering, a challenge is the mathematical intricacy introduced by time-delay modeling, potentially requiring advanced analytical techniques.

Reference [31] comprehensively explores stability analysis in neural networks, emphasizing dynamics from discrete and distributed delays. While offering insights into long-term stability properties, challenges arise from the mathematical intricacies of analyzing mixed delay conditions, potentially requiring specialized techniques. Article [32] intricately explores stability in recurrent neural networks, addressing complexities from multiple discrete and distributed delays. While offering insights into long-term behaviors, challenges emerge from the mathematical intricacies of analyzing networks with mixed delays, potentially requiring advanced techniques. Neural network dynamics with a novel framework addressing stability and bifurcation in networks with diverse delays is explored in Article [33]. While providing insights into stability transitions, challenges arise from the mathematical complexity of analyzing systems with mixed delays, potentially requiring specialized techniques.

The dynamics of nonlinear composite systems with distributed order dynamics and delays, introduce a novel asymptotic stability condition that considers uncertainties in fractional order explored in Article [34]. The method's advantages lie in handling fractional order uncertainties, enhancing its applicability, but potential challenges may arise from the mathematical complexity of analyzing systems with both distributed order and delays. Article [35] investigates the dynamics of nonlinear composite systems with distributed order dynamics and delays, presenting a novel asymptotic stability condition considering uncertainties in fractional order. The method excels in handling uncertainties in fractional order dynamics, extending its applicability to diverse fields. However, potential challenges may emerge from the mathematical complexity associated with analyzing systems involving both distributed order and delays, notwithstanding its substantial contributions to understanding stability in such composite systems. Reference [36] focuses on stability analysis in systems with distributed input delays using Lyapunov stability analysis. The method, known for its mathematical rigor, addresses scenarios prevalent in networked control systems and communication networks. Despite potential challenges related to the complexity of distributed input delays, the approach substantially contributes to understanding system stability and guides the design of resilient systems in practical applications. Article [37] delves into the stability of stochastic delayed recurrent neural networks, considering both discrete and distributed delays under stochastic influences. The method, utilizing advanced mathematical techniques, offers a comprehensive framework to assess network stability, applicable to machine learning and neuroscience. Despite potential challenges related to the mathematical complexity of stochastic processes and mixed delays, the approach contributes significantly to understanding and enhancing the design of resilient networks in practical applications.

In Table 1, our proposed method, as detailed in the article, undergoes a comprehensive comparison with other articles in the field published in recent years. This comparative analysis is grounded in the indexing of various characteristics intrinsic to these articles. Through this meticulous process, it becomes evident that our proposed method excels across all the considered indicators, underscoring the robustness and strength of the approach detailed in our article. The comparison highlights the unique and superior features of our method of contemporary works, solidifying its position as an impactful contribution to the field.

**Table 1:** Comparing this article and related works.

	Delay	Stability	Distributed	Nonlinear	Infinite	Gamma	Jensen Inequality	Uncertainty
[24]	✓	✓	✓					
[25]	✓	✓		✓				
[26]	✓	✓	✓		✓			
[27]	✓	✓	✓			✓		
[28]	✓		✓		✓			
[29]	✓	✓					✓	
[30]	✓	✓	✓					
[31]	✓	✓	✓					
[32]	✓	✓	✓					
[33]	✓	✓	✓					
[34]	✓		✓	✓				✓
[35]	✓		✓	✓				✓
[36]	✓	✓	✓					
[37]	✓	✓	✓					
This article	✓	✓	✓	✓	✓	✓	✓	✓

### 1.3 Research gaps and contributions

This research undertaking stands poised to address critical voids within the existing body of scholarly inquiry. While prior investigations have diligently probed the realms of stability analysis within delayed systems and gapped gamma distributions as distinct entities, their amalgamation in a unified framework remains an untrodden path. This union unveils novel challenges that, in turn, necessitate the development of specialized analytical tools to navigate the intricate terrain. It is within this context that the strategic amalgamation of augmented Lyapunov functions, Jensen's integral inequality, and LMIs emerges as a promising strategy to unlock the complex stability properties inherent to these intricate systems.

The primary contributions of this article can be succinctly summarized as follows.

• **Novel Stability Analysis:** Unveiling the stability properties of delayed systems entwined with gapped gamma distributions.
• **Augmented Lyapunov Functions:** Demonstrating the effectiveness of these functions in analyzing the stability of such complex systems.
• **Analytical Tools**: Applying Jensen's Inequality and LMIs for a comprehensive stability assessment.
• **Convergence and Asymptotic Stability:** Investigating convergence rates and asymptotic stability within the considered delayed system.
• **Complex System Dynamics:** Understanding the interdependence of gapped gamma distribution attributes and traffic flow model dynamics on stability.

### 1.4 Organization

The organizational framework for the remainder of this article unfolds as follows:

In Section 2, we delve into the mathematical foundation, introducing augmented Lyapunov functions and their relevance in stability analysis. Challenges in analyzing delayed systems with gapped gamma distributions are underscored. This section also provides a framework by outlining the utilization of Jensen's integral inequality and LMIs as part of our methodology. Moving on to Section 3, simulation results from stability analysis are presented, unveiling insights into system behavior. Lastly, in Section 4, the study culminates with a summary of key findings and a discussion of their wider implications across various scientific domains.

## 2 Problem formulation

In the Problem Formulation section, we aim to elucidate the details and objectives of the study on exponential stability in delayed systems influenced by gapped gamma distributions. This section serves as a crucial point to analyze and contextualize the multifaceted dimensions, unraveling the interplay between delayed system dynamics, gapped gamma distributions, and analytical tools like Jensen's integral inequality, LMIs, and Augmented Lyapunov functions. The synthesis bridges the gap between research objectives and methodologies, striving to establish a comprehensive stability analysis framework amidst the complexities of the loop traffic flow model.

The consideration of a gapped gamma distribution in the analysis of exponential stability is motivated by the need to model and understand more realistic delay scenarios in dynamical systems. The gapped gamma distribution provides a flexible framework for capturing diverse delay patterns that may exist in practical systems. The presence of gaps in the distribution allows for the representation of intermittent delays, which can be common in real-world scenarios.

In many applications, delays are not constant but exhibit variations over time, and the gapped gamma distribution offers a suitable mathematical description for such variability. By incorporating this distribution into the stability analysis, the study aims to address the complexities introduced by intermittent delays and provide a more accurate representation of the system's behavior. This consideration is essential for developing robust and reliable models that can better reflect the dynamics of systems subject to varying delay patterns, ultimately contributing to a more comprehensive understanding of stability in practical applications.

In exploring the exponential stability of delayed systems with split gamma distributions, we focus on the detailed analysis of system equation (1) and Kernel equation (2), considering that neither A nor $A_{0}$ adheres to the Horwitz criteria. This section outlines our investigative path, employing specialized analytical tools and methodologies, with a primary emphasis on utilizing an augmented Lyapunov function tailored to this context, serving as a crucial element in calculations and examinations leading to the presented results.

(1) $$\dot{x}\left(t\right)=Ax\left(t\right)+A_{d}\int_{0}^{\infty }\;\Gamma \left(\theta \right)x\left(t-\theta -\tau \right)d\theta$$

(2) $$\Gamma \left(\theta \right)=\frac{\theta^{N-1}{\underline{e}}^{-\frac{\theta }{T}}}{T^{N}\left(N-1\right)!}$$

When utilizing the kernel K(θ)=Γ(θ), the constants align with the expressions in equation (3), highlighting a straightforward relationship between the chosen kernel and the foundational parameters, simplifying their determination.

(3) $$\begin{array}{c} {\Gamma_{0\delta }≜K_{0\delta }|}_{K=\Gamma }=\int_{0}^{\infty }\;e^{2\delta \left(\theta +\tau \right)}\Gamma \left(\theta \right)d\theta =\frac{e^{2\delta \tau }}{{\left(1-2\delta T\right)}^{N}}\text{,}\\ {\Gamma_{1\delta }≜K_{1\delta }|}_{K=\Gamma }=\int_{0}^{\infty }\;e^{2\delta \left(\theta +\tau \right)}\Gamma \left(\theta \right)\left(\theta +\tau \right)d\theta =\\ \frac{e^{28\tau }}{{\left(1-2\delta T\right)}^{N}}\left(\tau +\frac{NT}{1-28T}\right),\Gamma_{10}=\tau +NT,\Gamma_{00}=1\text{.} \end{array}$$

Equation (4) is established, reinforcing a pivotal connection that contributes to the overarching framework of analysis, building a more comprehensive understanding of the system's dynamics.

(4) $$\int_{0}^{\infty }\;\Gamma \left(\theta \right)x\left(t-\theta -\tau \right)d\theta =\int_{-\infty }^{t}\;\Gamma \left(t-s\right)x\left(s-\tau \right)ds$$

In a delayed system governed by a gamma distribution with a gap and N=1, the system's dynamics align with the specified kernel in equation (5), leading to the validity of equation (6) and enhancing coherence in the analysis of the system's behavior within the framework of a gamma distribution with a gap.

(5) $$\Gamma \left(\theta \right)=\frac{e^{-\frac{\theta }{T}}}{T}$$

(6) $$\Gamma \left(0\right)=\frac{1}{T},\dot{\Gamma }\left(\theta \right)=-\frac{1}{T}\Gamma \left(\theta \right)$$

Introducing vector equation (7) as a pivotal definition capturing key variables [38], it serves as a cornerstone, enhancing the structural integrity of the analysis for a cohesive exploration of the intricate dynamics in the scrutinized system.

(7) $$y\left(t\right)=\int_{-\infty }^{t}\;\Gamma \left(t-s\right)x\left(s-\tau \right)ds$$

Transforming system equation (8) into the adjoint form equation (9) provides a significant stride, revealing interrelatedness and offering fresh insights into the system's dynamics, enabling a more comprehensive analysis for a deeper understanding.

(8) $$\dot{x}\left(t\right)=Ax\left(t\right)+A_{d}\int_{0}^{\infty }\;\Gamma \left(\theta \right)x\left(t-\theta -\tau \right)d\theta$$

(9) $$\dot{x}\left(t\right)=Ax\left(t\right)+A_{d}y\left(t\right),\dot{y}\left(t\right)=\frac{1}{T}x\left(t-\tau \right)-\frac{1}{T}y\left(t\right)$$

The stability of the supplementary system impacts the primary system, emphasizing the non-reciprocal nature, akin to model transformation in systems with discrete delays, revealing intricate interplay within delayed systems.

In the context of the loop traffic flow model, for τ=0, the system matrix exhibits a zero eigenvalue, revealing a nuanced aspect challenging the direct applicability of prior results due to non-conformity with Horwitz's equation (10).

(10) $$A_{0}=A+A_{d}\int_{0}^{\infty }\;\Gamma \left(\theta \right)d\theta$$

This article aims to comprehensively analyze the system's exponential stability, focusing on cases where the traditional Lyapunov function is not applicable for τ=0, providing insights into the limitations of conventional analytical tools within equation (11).

(11) $$V_{\text{aug}\;}\left(t\right)=\left[\begin{array}{c} x^{T}\left(t\right)\;y^{T}\left(t\right) \end{array}\right]\left[\begin{array}{c} P\;Q\\ {}*\;Z \end{array}\right]\left[\begin{array}{c} x\left(t\right)\\ y\left(t\right) \end{array}\right],\left[\begin{array}{c} P\;Q\\ {}*\;Z \end{array}\right]>0$$

To rigorously scrutinize the exponential stability inherent in system (8), we introduce an innovative analytical approach. This entails the proposal of an additional Lyapunov function, denoted as equation (12). By incorporating this supplementary Lyapunov function into our analysis, we aim to enhance our understanding of the stability dynamics intrinsic to the system. This pioneering method enables a more comprehensive exploration of the system's behavior, illuminating nuances that may otherwise elude traditional stability assessment techniques. Through the incorporation of this additional Lyapunov function, we seek to unveil insights that contribute to the broader comprehension of the system's exponential stability.

(12) $$\begin{array}{c} V\left(t\right)=V_{\text{aug}\;}\left(t\right)+V_{G}\left(t\right)+V_{H}\left(t\right)+V_{S}\left(t\right)+V_{R}\left(t\right)\text{,}\\ V_{G}\left(t\right)=\int_{0}^{\infty }\;\int_{t-\theta -\tau }^{t}\;e^{-2\delta \left(t-s\right)}\Gamma \left(\theta \right)x^{T}\left(s\right)Gx\left(s\right)dsd\theta \text{,}\\ V_{H}\left(t\right)=\int_{0}^{\infty }\;\int_{0}^{\theta +\tau }\;\int_{t-\lambda }^{t}\;e^{-2\delta \left(t-s\right)}\Gamma \left(\theta \right){\dot{x}}^{T}\left(s\right)H\dot{x}\left(s\right)dsd\lambda d\theta G>0,H>0,R>0,S>0\text{.}\\ V_{S}\left(t\right)=\int_{t-\tau }^{t}\;e^{-2\delta \left(t-s\right)}x^{T}\left(s\right)Sx\left(s\right)ds\text{,}\\ V_{R}\left(t\right)=\int_{-\tau }^{0}\;\int_{t+\theta }^{t}\;e^{-2\delta \left(t-s\right)}{\dot{x}}^{T}\left(s\right)R\dot{x}\left(s\right)dsd\theta \text{,} \end{array}$$

Equation (13) is derived through a meticulous process involving the adjoint system and the augmented Lyapunov function $V_{aug}$, serving as a significant bridge that enhances the comprehension of the system's stability characteristics by capturing the intricate interplay between system components and their relationships.

The rationale for opting for augmented LKF can be succinctly outlined as follows.

1. Handling Intermittent Delays: Traditional Lyapunov functions may struggle to capture the effects of intermittent delays, especially when dealing with complex delay patterns like those modeled by gapped gamma distributions. Augmented LKF allows for a more flexible representation, accommodating the intermittent nature of delays and providing a nuanced analysis.
2. Improved Stability Analysis: Augmented LKF offers an extended framework that includes additional terms to compensate for the complexities introduced by the gapped gamma distribution. This allows for a more comprehensive stability analysis, particularly in scenarios where traditional Lyapunov functions may fall short.
3. Enhanced Realism in Modeling: The use of augmented LKF aligns with the need to enhance the realism of our model. By incorporating additional terms into the Lyapunov functions, we can better capture the intricate dynamics associated with gapped gamma distribution, leading to more accurate stability assessments.

In summary, the choice of augmented LKF is motivated by its ability to overcome limitations associated with traditional Lyapunov functions in the context of delayed systems with gapped gamma distribution. This decision aims to provide a more robust and accurate analytical tool for studying the stability of such complex dynamical systems.

(13) $$\begin{array}{c} {\dot{V}}_{aug}\left(t\right)=2\left[x^{T}\left(t\right)y^{T}\left(t\right)\right]\left[\begin{array}{cc} P & Q\\ {}* & Z \end{array}\right]\left[\begin{array}{c} \dot{x}\left(t\right)\\ \dot{y}\left(t\right) \end{array}\right]=2\eta^{T}\left(t\right)\left[\begin{array}{ccc} PA & PA_{d}-\frac{1}{T}Q & \frac{1}{T}Q\\ Q^{T}A & Q^{T}A_{d}-\frac{1}{T}Z & \frac{1}{T}Z\\ 0 & 0 & 0 \end{array}\right]\eta \left(t\right)\text{.}\\ \eta \left(t\right)=\text{col}\left\{x\left(t\right),y\left(t\right),x\left(t-\tau \right)\right\};= \end{array}$$

Leveraging the potency of Jensen's integral inequality, our analysis takes a decisive turn as we delve into the exploration of equation (3) with N=1. Through this analytical endeavor, we achieve the establishment of equation (14). The utilization of Jensen's integral inequality, a venerable mathematical tool renowned for its capacity to bind non-linear terms, facilitates the derivation of these crucial relationships. This analytical feat enhances our understanding of the intricate interconnections within the system, unearthing insights that resonate with the interplay of delayed dynamics and distributional characteristics. As we navigate through this mathematical landscape, the establishment of equation (14) stands as a testament to the analytical power wielded by the application of Jensen's integral inequality within our investigation.

(14) $$\begin{array}{c} {\dot{V}}_{S}\left(t\right)+{\dot{V}}_{G}\left(t\right)+2\delta \left[V_{S}\left(t\right)+V_{G}\left(t\right)\right]\leq \\ x^{T}\left(t\right)\left[S+G\right]x\left(t\right)-e^{-2\delta \tau }x^{T}\left(t-\tau \right)Sx\left(t-\tau \right)-\Gamma_{0\delta }^{-1}\int_{0}^{\infty }\;\Gamma \left(\theta \right)x^{T}\left(t-\theta -\tau \right)d\theta G\int_{0}^{\infty }\;\Gamma \left(\theta \right)x\left(t-\theta -\tau \right)d\theta \text{,}\\ {\dot{V}}_{R}\left(t\right)+{\dot{V}}_{H}\left(t\right)+2\delta \left[V_{R}\left(t\right)+V_{H}\left(t\right)\right]\leq \\ {\dot{x}}^{T}\left(t\right)\left[\tau R+\left(\tau +T\right)H\right]\dot{x}\left(t\right)-\frac{e^{-2\delta \tau }}{\tau }\int_{t-\tau }^{t}\;{\dot{x}}^{T}\left(s\right)dsR\int_{t-\tau }^{t}\;\dot{x}\left(s\right)ds-\Gamma_{18}^{-1}\int_{0}^{\infty }\;\int_{t-\theta -\tau }^{t}\;\Gamma \left(\theta \right){\dot{x}}^{T}\left(s\right)dsd\theta H\int_{0}^{\infty }\;\int_{t-\theta -\tau }^{t}\;\Gamma \left(\theta \right)\dot{x}\left(s\right)dsd\theta \text{.} \end{array}$$

In the field of stability analysis for systems with time delays, various integral inequalities have been developed, some of which are less conservative than Jensen's Integral Inequality. Notable examples include.

1 Reference [39]:
  • **Approach:** This study, introduces delay-derivative-dependent slack matrices that provide additional freedom to optimize Lyapunov matrices, resulting in less conservative stability conditions based on the Lyapunov-Krasovskii functional approach.
  • **Comparison:** While this approach allows for a more flexible stability analysis, Jensen's Integral Inequality provides a straightforward method for deriving stability conditions without the added complexity of delay-derivative dependencies.
  • **Discussion:** The flexibility offered by delay-derivative-dependent slack matrices can lead to more precise stability bounds. However, Jensen's Integral Inequality remains a robust choice for simpler systems or when computational resources are limited.
2. Article [40]:
  F0B7**Approach**: This paper addresses the absolute stability of uncertain Lur'e systems using a delay-segmentation approach, decomposing the delay interval into subintervals, which allows for an augmented Lyapunov–Krasovskii functional that ensures piecewise continuity.
  F0B7**Comparison**: The delay-segmentation approach offers a fine-grained analysis, particularly useful for systems with varying delays, whereas Jensen's Integral Inequality provides a more generalized stability criterion that can be applied without segmenting delays.
  F0B7**Discussion**: While the delay-segmentation method can yield less conservative results by tailoring the stability analysis to specific delay intervals, Jensen's Integral Inequality offers a balance between simplicity and effectiveness for a broad range of delayed systems.
3. Approach [41]:
  F0B7**Approach**: This study presents a generalized free-matrix-based integral inequality (GFMBII) that deals with time-varying delay systems without using the reciprocal convexity lemma, leading to improved stability criteria.
  F0B7**Comparison**: The GFMBII approach avoids the limitations of the reciprocal convexity lemma, offering more accurate stability bounds. In contrast, Jensen's Integral Inequality is simpler to implement but might be more conservative in some scenarios.
  F0B7**Discussion**: The GFMBII method's ability to handle time-varying delays with greater precision is advantageous for complex systems. However, Jensen's Integral Inequality remains a valuable tool for initial stability assessments and for systems where ease of implementation is a priority.

Our research incorporates Jensen's Integral Inequality and LMIs in the stability analysis of delayed systems with gapped gamma distributions, using an Augmented Lyapunov Function. While the aforementioned methods offer less conservative stability conditions, Jensen's Integral Inequality provides a solid foundation for deriving stability criteria that are both effective and computationally efficient. By acknowledging these less conservative methods, we highlight the trade-offs between analytical precision and computational complexity, ensuring that our approach remains relevant and applicable within the broader context of stability analysis.

Consequently, the discourse evolves to encompass inequality (15), which is a core of exponential stability analysis. Intriguingly, this inequality attains significance in the presence of LMI (16). The interrelation between these two components is pivotal, as it underscores the delicate balance between stability assessment and mathematical formalism. In essence, the validity of inequality equation (15), emblematic of exponential stability, is intricately linked to adherence to LMI equation (16). This convergence of concepts signifies a fundamental alignment, where mathematical expressions and stability considerations interweave to provide a holistic comprehension of the system's behavior. Through this pivotal relationship, we navigate the intricate path toward deducing the system's stability dynamics.

(15) $$\dot{V}\left(t\right)+2\delta V\leq 0$$

(16) $$\begin{array}{c} \left[\begin{array}{cccc} \Psi_{11} & \Psi_{12} & \frac{1}{T}Q+\frac{e^{-2\delta \tau }}{\tau }R & A^{T}\left[\tau R+\left(\tau +T\right)H\right]\\ {}* & \Psi_{22} & \frac{1}{T}Z^{-28} & A_{d}^{T}\left[\tau R+\left(\tau +T\right)H\right]\\ {}* & * & -e^{-2\delta \tau }S-\frac{e^{-2\delta \tau }}{\tau }R & 0\\ {}* & * & * & -\tau R-\left(\tau +T\right)H \end{array}\right]<0\\ \Psi_{11}=PA+A^{T}P+2\delta P+S+G-\frac{e^{-2\delta \tau }}{\tau }R-\Gamma_{18}^{-1}H\\ \Psi_{12}=PA_{d}-\frac{1}{T}Q+A^{T}Q+\Gamma_{1\delta }^{-1}H+2\delta Q\\ \Psi_{22}=Q^{T}A_{d}+A_{d}^{T}Q-\frac{2}{T}Z-\Gamma_{0\delta }^{-1}G-\Gamma_{18}^{-1}H+2\delta Z \end{array}$$

For the scenario where τ=0, we pivot our analytical approach by adopting the Lyapunov function $V\left(t\right)=V_{aug}\left(t\right)+V_{G}\left(t\right)+V_{H}\left(t\right)$. Here, the constituents $V_{aug}\left(t\right)$, $V_{G}\left(t\right)$, and $V_{H}\left(t\right)$ are derived using the same preceding equations, albeit with τ set to 0. This strategic shift allows us to unravel insights pertinent to this specific temporal condition. Intriguingly, this approach leads us to the derivation of the corresponding LMI, encapsulated within equation (17). This LMI serves as a linchpin in our analysis, embodying the intricate relationship between stability assessment and mathematical formulations. Through the embodiment of these principles, we navigate the complexities of stability analysis under the specific parameterization of τ=0, yielding insights that illuminate the system's behavior within this temporal context.

(17) $$\begin{array}{c} \left[\begin{array}{ccc} \Psi_{11} & \Psi_{12}TA^{T}H & \\ {}* & \Psi_{22} & TA_{d}^{T}H\\ {}* & * & -TH \end{array}\right]<0\\ \Psi_{11}=PA+A^{T}P+2\delta P+G+\frac{1}{T}\left(Q+Q^{T}\right)-\Gamma_{1\delta^{-1}}H\\ \Psi_{12}=PA_{d}-\frac{1}{T}Q+A^{T}Q+\frac{1}{T}Z+2\delta Q+\Gamma_{1\delta }^{-1}H\\ \Psi_{22}=Q^{T}A_{d}+A_{d}^{T}Q-\frac{2}{T}Z+2\delta Z-\Gamma_{0\delta }^{-1}G-\Gamma_{1\delta }^{-1}H\text{.} \end{array}$$

Result 1 illuminates a pivotal finding within our analysis, centering on the stability dynamics of the system equation (18). This system, characterized by a Gamma distributed kernel equation (19) and N=1, assumes paramount significance. Notably, the condition for exponential stability arises when the convergence rate, denoted as δ and constrained within the interval δ∈(0,1/T), adheres to specific criteria. This outcome encapsulates a nuanced interplay between system parameters and the imposed constraints on the convergence rate, offering insights that underscore the intricate balancing act between temporal dynamics and stability considerations. The convergence rate δ, nestled within this critical range, emerges as a determining factor that influences the system's exponential stability, delineating boundaries within which the system behavior remains tractable and predictable.

(18) $$\dot{x}\left(t\right)=Ax\left(t\right)+A_{d}\int_{0}^{\infty }\;\Gamma \left(\theta \right)x\left(t-\theta -\tau \right)d\theta$$

(19) $$\Gamma \left(\theta \right)=\frac{\theta^{N-1}{\underline{e}}^{-\frac{\theta }{T}}}{T^{N}\left(N-1\right)!}$$

1. For τ>0, a compelling revelation surfaces: the existence of positive definite matrices $P,R,S,G,H,Z\in R^{n\times n}$, along with a matrix $Q\in R^{n\times n}$, coalesce to establish a series of LMIs, as delineated by equation (20). This observation underscores a critical juncture in our analysis, wherein these matrices collectively imbue the system with a mathematical framework that substantiates our understanding of stability dynamics. These LMIs serve as core elements, encapsulating the intricate interplay of matrices and inequalities that define the system's stability characteristics under the temporal parameter τ>0. This remarkable achievement embodies the synergy between mathematical formalism and stability analysis, marking a crucial stride toward unraveling the stability properties of the system within this temporal context.

(20) $$\left[\begin{array}{cccc} \Psi_{11} & \Psi_{12} & \frac{1}{T}Q+\frac{e^{-2\delta \tau }}{\tau }R & A^{T}\left[\tau R+\left(\tau +T\right)H\right]\\ {}* & \Psi_{22} & \frac{1}{T}Z^{-28} & A_{d}^{T}\left[\tau R+\left(\tau +T\right)H\right]\\ {}* & * & -e^{-2\delta \tau }S-\frac{e^{-2\delta \tau }}{\tau }R & 0\\ {}* & * & * & -\tau R-\left(\tau +T\right)H \end{array}\right]<0,\left[\begin{array}{cc} P & Q\\ {}* & Z \end{array}\right]>0$$

2. When τ=0, a significant insight emerges: the presence of positive definite matrices $P,G,H,Z\in R^{n\times n}$, alongside a matrix $Q\in R^{n\times n}$, converge to establish a series of LMIs as articulated in equation (21). This observation underscores a pivotal juncture in our investigation, wherein these matrices collectively form a mathematical scaffold that substantiates our comprehension of stability dynamics. These LMIs stand as fundamental components, encapsulating the intricate interplay between matrices and inequalities that underpin the system's stability traits under τ=0. This achievement exemplifies the symbiosis between mathematical rigor and stability analysis, marking a decisive step forward in disentangling the stability attributes of the system within this temporal context.

(21) $$\left[\begin{array}{ccc} \Psi_{11} & \Psi_{12}TA^{T}H & \\ {}* & \Psi_{22} & TA_{d}^{T}H\\ {}* & * & -TH \end{array}\right]<0,\left[\begin{array}{cc} P & Q\\ {}* & Z \end{array}\right]>0$$

If LMIs remain valid for δ = 0, an implication arises: the system achieves exponential stability with a small convergence rate, showcasing the nuanced interplay between stability and convergence, particularly evident in equation (22) within scenarios involving gamma distribution delays with a gap and N≥2. This underscores the intricate relationship between delay distribution, system characteristics, and stability under specific temporal configurations.

(22) $$\Gamma \left(0\right)=0,\dot{\Gamma }\left(\theta \right)=-\frac{1}{T}\cdot \Gamma \left(\theta \right)+g\left(\theta \right),g\left(\theta \right)=\frac{\theta^{N-2}e^{-\frac{\theta }{T}}}{T^{N}\left(N-2\right)!}$$

In the context of this specific delay scenario, our analysis pivots to consider a Lyapunov function denoted as equation (23). This strategic shift reflects our intent to delve into the stability attributes under the distinctive delay configuration at hand. By introducing this Lyapunov function, we aim to glean insights that unravel the system's behavior within the framework of this particular delay type. This choice of the Lyapunov function serves as a core, facilitating a more targeted exploration of the system's stability characteristics while accounting for the unique temporal dependencies inherent to this delay configuration.

(23) $$\begin{array}{c} V\left(t\right)=V_{aug}\left(t\right)+V_{G}+V_{H}+V_{E}+V_{F}\\ V_{aug}\left(t\right)=\left[x^{T}\left(t\right)y^{T}\left(t\right)\right]\left[\begin{array}{cc} P & Q\\ {}* & Z \end{array}\right]\left[\begin{array}{c} x\left(t\right)\\ y\left(t\right) \end{array}\right]\\ V_{G}\left(t\right)=\int_{0}^{\infty }\;\int_{t-\theta -\tau }^{t}\;e^{-2\delta \left(t-s\right)}\Gamma \left(\theta \right)x^{T}\left(s\right)Gx\left(s\right)dsd\theta \text{,}\\ V_{H}\left(t\right)=\int_{0}^{\infty }\;\int_{0}^{\theta +\tau }\;\int_{t-\lambda }^{t}\;e^{-2\delta \left(t-s\right)}\Gamma \left(\theta \right){\dot{x}}^{T}\left(s\right)H\dot{x}\left(s\right)dsd\lambda d\theta \\ V_{E}\left(t\right)=\int_{0}^{\infty }\;\int_{t-\theta -\tau }^{t}\;e^{-2\delta \left(t-s\right)}g\left(\theta \right)x^{T}\left(s\right)Ex\left(s\right)dsd\theta ,E>0\;\text{and}\;F>0\\ V_{F}\left(t\right)=\int_{0}^{\infty }\;\int_{0}^{\theta +\tau }\;\int_{t-\lambda }^{t}\;e^{-2\delta \left(t-s\right)}g\left(\theta \right){\dot{x}}^{T}\left(s\right)F\dot{x}\left(s\right)dsd\lambda d\theta \end{array}$$

In this context, a nuanced strategy unfolds, involving the incorporation of two distinct terms, namely $V_{F}\left(t\right)$ and $V_{E}\left(t\right)$. These additions are strategically orchestrated to counterbalance the impact posed by the presence of term equation (24) within the augmented Lyapunov function ${\dot{V}}_{aug}$. This approach reflects a nuanced mitigation strategy, wherein the introduction of $V_{F}\left(t\right)$ and $V_{E}\left(t\right)$ serves to neutralize the influence of term equation (24), thus enhancing the stability analysis framework. This strategic maneuver underscores the intricacies inherent to the analysis, where deliberate adjustments are introduced to foster a more accurate and comprehensive understanding of the system's stability attributes.

(24) $$\int_{-\infty }^{t}\;g\left(t-\theta \right)x^{T}\left(\theta -\tau \right)d\theta$$

By embarking on a process of derivation and definition, a pivotal milestone is reached with the establishment of constants equation (25). These constants assume a critical role in the analysis, underpinning the subsequent attainment of a sufficient LMI as delineated in result (2). This achievement underscores a fundamental linkage between the derived constants and the ensuing stability analysis, highlighting the interconnectedness between mathematical formulations and stability considerations. Through the meticulous derivation and definition of these constants, the groundwork is laid for the emergence of a robust LMI, thus advancing our comprehension of the system's stability attributes within this analytical framework.

(25) $$\begin{array}{c} G_{0\delta }={K_{0\delta }|}_{K=g}=\int_{0}^{\infty }\;e^{2\delta \left(\theta +\tau \right)}g\left(\theta \right)d\theta =e^{2\delta \tau }\frac{1}{T{\left(1-2\delta T\right)}^{N-1}}\text{,}\\ G_{1\delta }={K_{1\delta }|}_{K=g}=e^{2\delta \tau }\frac{N-1+\frac{\tau }{T}\left(1-2\delta T\right)}{{\left(1-2\delta T\right)}^{N}},G_{00}=\frac{1}{T},G_{10}=N-1+\frac{\tau }{T} \end{array}$$

Result 2 stands as a pivotal culmination, unfolding within the confines of the specified parameter range δ∈(0,1/T) with a particular focus on the instance where δ=0. In this scenario, a key observation emerges: the existence of positive definite matrices $P,E,F,G,H,Z\in R^{n\times n}$, alongside a matrix $Q\in R^{n\times n}$, manifests in a manner that aligns with the establishment of a series of LMIs, as delineated in equation (26). This outcome underscores a pivotal juncture in our analytical pursuit, wherein the interplay of matrices, inequalities, and stability considerations converges to forge a path toward comprehending the system's stability characteristics. The interplay between these matrices and LMIs serves as a testament to the intricate balance between mathematical formalism and the exploration of stability dynamics, highlighting the foundational role of these elements in our analytical article.

(26) $$\begin{array}{c} \left[\begin{array}{cc} P & Q\\ {}* & Z \end{array}\right]>0,\left[\begin{array}{cccc} \Xi_{11} & \Xi_{12} & Q+\frac{G_{00}}{G_{1\delta }}F & A^{T}\left(G_{10}F+\Gamma_{10}H\right)\\ {}* & \Xi_{22} & Z & A_{d}^{T}\left(G_{10}F+\Gamma_{10}H\right)\\ {}* & * & -G_{0\delta }^{-1}E-G_{1\delta }^{-1}F & 0\\ {}* & * & * & -G_{10}F-\Gamma_{10}H \end{array}\right]<0\\ \Xi_{11}=PA+A^{T}P+2\delta P+\frac{1}{T}E+G-\frac{G_{00}^{2}}{G_{1\delta }}F-\frac{1}{\Gamma_{1\delta }}H,\\ \Xi_{12}=PA_{d}-\frac{1}{T}Q+A^{T}Q+2\delta Q+\frac{1}{\Gamma_{18}}H,\\ \Xi_{22}=Q^{T}A_{d}+A_{d}^{T}Q-\frac{2}{T}Z+2\delta Z-\frac{1}{\Gamma_{08}}G-\frac{1}{\Gamma_{1\delta }}H.\; \end{array}$$

In this particular scenario, a significant observation emerges: the system attains a state of exponential stability, characterized by a convergence rate δ. Notably, this stability is realized under the condition of a sufficiently small convergence rate, underscoring the delicate balance between stability considerations and the rate at which the system converges. It's noteworthy that for instances where N≥2, systems characterized by distributed delay gamma distributions can be transformed into an additive system featuring a discrete delay τ. To this end, the Gamma distributed kernel, denoted as $\Gamma_{N}\left(\theta \right)$, assumes prominence and equation (27) comes into play as a central component of this transformation. This observation unveils the intricate interplay between system characteristics, delay configurations, and stability dynamics, showcasing the potential for transformative insights within the context of complex systems.

(27) $${\dot{\Gamma }}_{N}\left(\theta \right)=\frac{1}{T}\Gamma_{N-1}\left(\theta \right)-\frac{1}{T}\Gamma_{N}\left(\theta \right)$$

Following a meticulous analytical path, we establish an adjoint system with a state vector in equation (28), showcasing a strategic alignment that enables a comprehensive analysis of stability characteristics. This pivotal advancement underscores our commitment to unraveling system dynamics intricacies, providing deeper insights into the interplay between components and behavior.

(28) $$\eta_{N}\left(t\right)=\text{col}\;\left\{x\left(t\right),\int_{0}^{\infty }\;\Gamma_{N}\left(\theta \right)x\left(t-\theta -\tau \right)d\theta ,\ldots ,\int_{0}^{\infty }\;\Gamma_{1}\left(\theta \right)x\left(t-\theta -\tau \right)d\theta \right\}$$

In the pursuit of reduced conservatism in LMIs, a strategic move is made, introducing vector equation (29) within N = 2, aiming to balance computational efficiency and accuracy. This analytical choice navigates the complex terrain of stability analysis, acknowledging the trade-offs between conservatism reduction and increased computational demands.

(29) $$\xi \left(t\right)=\int_{-\infty }^{t}\;g\left(t-\theta \right)x\left(\theta -\tau \right)d\theta$$

Within this context, a notable transition occurs: the response of the original system, as characterized by equation (30), is seamlessly extended to encompass the scope of equation (31). This strategic extension enables us to bridge the gap between the original system's behavior and its implications within the framework of equation (31). Through this alignment, we endeavor to gain a deeper insight into the dynamics of the system and its response to the conditions posed by equation (31). This critical link between system behavior and the implications of equation (31) embodies the essence of our analytical pursuit, where mathematical formalism converges with system dynamics to shed light on the intricacies of stability analysis within this specific context.

(30) $$\dot{x}\left(t\right)=Ax\left(t\right)+A_{d}\int_{0}^{\infty }\;\Gamma \left(\theta \right)x\left(t-\theta -\tau \right)d\theta$$

(31) $$\dot{x}\left(t\right)=Ax\left(t\right)+A_{d}y\left(t\right),\dot{y}\left(t\right)=-\frac{1}{T}y\left(t\right)+\xi \left(t\right),\dot{z}\left(t\right)=\frac{1}{T^{2}}x\left(t-\tau \right)-\frac{1}{T}z\left(t\right)$$

Within this realm of analysis, a fundamental observation surfaces: when matrices A and $A_{d}$ assume the state of singularity, the aspiration for achieving asymptotic stability becomes unattainable. This revelation underscores the significance of the system's inherent characteristics, specifically the singular nature of matrices A and $A_{d}$, which collectively thwart the pursuit of asymptotic stability. Within this framework, a Lyapunov function emerges as a pivotal tool, characterized by the formulation denoted as equation (32). This strategic choice of Lyapunov function encapsulates our intent to navigate the complexities of the system's stability dynamics, albeit within the constraints imposed by the singular nature of matrices A and $A_{d}$. This mathematical foundation serves as a beacon, illuminating our quest to understand stability attributes even within scenarios where the conventional pathway to asymptotic stability remains obstructed.

(32) $$V_{aug}=\eta_{2}^{T}\left(t\right)P\eta_{2}\left(t\right)\;\text{and}\;0<P\in R^{3n\times 3n}$$

The adoption of the Lyapunov function (equation (32)) leads to the formulation of a pivotal LMI (equation (33)), serving as a mathematical core that intricately captures the interplay between matrices, inequalities, and stability attributes in the analytical framework. This achievement underscores the profound synergy between mathematical rigor and exploring stability dynamics, marking a decisive step forward in comprehending the interplay of system components and their stability attributes.

$$\left[\begin{array}{ccccc} \Lambda_{11} & \Lambda_{12} & \Lambda_{13} & \Lambda_{14} & \Lambda_{15}\\ {}* & \Lambda_{22} & \Lambda_{23} & \frac{1}{T^{2}}Q_{3} & \Lambda_{25}\\ {}* & * & \Lambda_{33} & \frac{1}{T^{2}}P_{3} & 0\\ {}* & * & * & \Lambda_{44} & 0\\ {}* & * & * & * & \Lambda_{55} \end{array}\right]<0,P=\left[\begin{array}{ccc} P_{1} & Q_{1} & Q_{2}\\ {}* & P_{2} & Q_{3}\\ {}* & * & P_{3} \end{array}\right]>0$$

(33) $$\begin{array}{c} \Lambda_{11}=P_{1}A+A^{T}P_{1}+S-\frac{e^{-2\delta \tau }}{\tau }R+2\delta P_{1}+G_{00}E+\Gamma_{00}G-\frac{G_{00}^{2}}{G_{1\delta }}F-\frac{\Gamma_{00}^{2}}{\Gamma_{18}}H\\ \Lambda_{12}=P_{1}A_{d}+A^{T}Q_{1}-\frac{1}{T}Q_{1}+2\delta Q_{1}+\frac{\Gamma_{00}}{\Gamma_{1\delta }}H\\ \Lambda_{13}=A^{T}Q_{2}+Q_{1}-\frac{1}{T}Q_{2}+\frac{G_{00}}{G_{1\delta }}F+2\delta Q_{2}\text{,}\\ \Lambda_{22}=Q_{1}^{T}A_{d}+A_{d}^{T}Q_{1}-\frac{2}{T}P_{2}+2\delta P_{2}-\frac{1}{\Gamma_{0\delta }}G-\frac{1}{\Gamma_{1\delta }}H\\ \Lambda_{23}=A_{d}^{T}Q_{2}+P_{2}-\frac{2}{T}Q_{3}+2\delta Q_{3}\text{,}\\ \Lambda_{33}=-\frac{2}{T}P_{3}+Q_{3}+Q_{3}^{T}-\frac{1}{G_{08}}E-\frac{1}{G_{18}}F+2\delta P_{3}\text{,}\\ \Lambda_{14}=\frac{1}{T^{2}}Q_{2}+\frac{e^{-2\delta \tau }}{\tau }R,\Lambda_{44}=-e^{-2\delta \tau }\left(S+\frac{1}{\tau }R\right)\text{,}\\ \Lambda_{55}=-\left(\tau R+\Gamma_{10}H+G_{10}F\right)\\ \Lambda_{15}=-A^{T}\Lambda_{55},\Lambda_{25}=-A_{d}^{T}\Lambda_{55}\text{.} \end{array}$$

A noteworthy observation emerges from the outcomes within this section: the resulting LMIs bear an affinity to the matrices A and $A_{d}$ ($A_{d1},\ldots ,A_{dm}$). This connection underscores a significant insight when the system matrices are associated with an indeterminate polytope, the analytical endeavor is streamlined. Specifically, the solution to the LMIs need only be pursued within the corners of the polytope. This strategic maneuver capitalizes on the structural characteristics of the system matrices, enabling a more focused analysis that is sensitive to the intricacies of the polytope's corners.

Furthermore, within the scope of finite delays and finite exponential kernels, an additional avenue emerges to enhance the analytical outcomes. This augmentation is accomplished through the introduction of supplementary functions that contribute to refining the analysis and enriching the results. Within this analytical backdrop, the spotlight shifts to the system articulated as equation (34), featuring finite delay h<∞ and, for the sake of simplicity, τ=0. This strategic consideration underscores our commitment to uncovering pathways that enhance the analysis's accuracy and effectiveness, showcasing the multidimensional nature of stability analysis within complex temporal configurations.

(34) $$\dot{x}\left(t\right)=Ax\left(t\right)+A_{d}\int_{0}^{h}\;\Gamma \left(\theta \right)x\left(t-\theta \right)d\theta ,\Gamma \left(\theta \right)=\frac{1}{T}e^{-\frac{\theta }{T}}$$

Leveraging definition equation (35) strategically, our analytical framework gains a fundamental tool, facilitating a nuanced exploration of the system's dynamics and stability attributes. This strategic deployment underscores a commitment to employing diverse mathematical tools for a comprehensive understanding of the system's behavior.

(35) $$y\left(t\right)=\int_{0}^{h}\;\Gamma \left(\theta \right)x\left(t-\theta \right)d\theta =\int_{t-h}^{t}\;\Gamma \left(t-s\right)x\left(s\right)ds$$

Through a strategic transformation, the interplay between the system articulated (34) and the essence of the definition (35) takes a decisive turn. This transformative maneuver leads us to the emergence of an adjoint system, characterized by a discrete delay, as encapsulated within equation (36). This transformation underscores our intent to bridge the gap between the original system's behavior and its adjoint counterpart, facilitating a more comprehensive analysis of stability attributes. Through this transition, we delve into the interplay between system components, discrete delays, and stability dynamics, thereby unraveling insights that resonate with the broader system behavior and its response to discrete temporal configurations.

$$\dot{x}\left(t\right)=Ax\left(t\right)+A_{d}y\left(t\right)\text{,}$$

(36) $$\dot{y}\left(t\right)=\frac{1}{T}\left[x\left(t\right)-y\left(t\right)-e^{-\frac{h}{T}}x\left(t-h\right)\right]$$

In a parallel manner to the scenario when h=∞, a noteworthy observation arises: the singularity of matrices A and $A_{0}$ may influence the asymptotic stability of the adjoint system, even as the original system retains its state of exponential stability. Within this intriguing dynamic, the strategic introduction of a Lyapunov function assumes prominence. This Lyapunov function, elegantly captured by equation (37), is strategically proposed to navigate the intricacies of this scenario. By leveraging this Lyapunov function, we aim to delineate stability attributes that reflect the delicate interplay between singular matrices, system behavior, and the prospect of exponential stability within the original system. This choice of the Lyapunov function serves as a mathematical core, guiding us toward a deeper understanding of stability dynamics within this specific context.

(37) $$\begin{array}{c} \bar{V}\left(t\right)=V_{\text{aug}\;}\left(t\right)+{\bar{V}}_{G}\left(t\right)+{\bar{V}}_{H}\left(t\right)+{\bar{V}}_{R}\left(t\right)+{\bar{V}}_{S}\left(t\right)\\ V_{\text{aug}\;}\left(t\right)=\left[\begin{array}{cc} x^{T}\left(t\right) & y^{T}\left(t\right) \end{array}\right]\left[\begin{array}{cc} P & Q\\ {}* & Z \end{array}\right]\left[\begin{array}{c} x\left(t\right)\\ y\left(t\right) \end{array}\right]\\ {\bar{V}}_{G}\left(t\right)=\int_{0}^{h}\;\int_{t-\theta }^{t}\;e^{-2\delta \left(t-s\right)}\Gamma \left(\theta \right)x^{T}\left(s\right)Gx\left(s\right)dsd\theta \text{,}\\ {\bar{V}}_{H}\left(t\right)=\int_{0}^{h}\;\int_{0}^{\theta }\;\int_{t-\lambda }^{t}\;e^{-2\delta \left(t-s\right)}\Gamma \left(\theta \right){\dot{x}}^{T}\left(s\right)H\dot{x}\left(s\right)dsd\lambda d\theta ,\\ {\bar{V}}_{S}\left(t\right)=\int_{t-h}^{t}\;e^{-2\delta \left(t-s\right)}x^{T}\left(s\right)Sx\left(s\right)ds,\\ {\bar{V}}_{R}\left(t\right)=\int_{-h}^{0}\;\int_{t+\theta }^{t}\;e^{-2\delta \left(t-s\right)}{\dot{x}}^{T}\left(s\right)R\dot{x}\left(s\right)dsd\theta \end{array}$$

Strategically defining constants as equation (38) leads to a significant milestone, the attainment of result (3), revealing the delicate interplay between mathematical formalism and analytical outcomes. This underscores a commitment to deriving insights for a nuanced understanding of the system's behavior and stability within the defined constants.

(38) $$\begin{array}{c} \Gamma_{00}^{h}=\int_{0}^{h}\;\Gamma \left(\theta \right)d\theta =1-e^{-\frac{h}{T}},\Gamma_{0\delta }^{h}=\int_{0}^{h}\;e^{2\delta \theta }\Gamma \left(\theta \right)d\theta =\frac{e^{\left(28-\frac{1}{T}\right)h}-1}{2\delta T-1}\\ \Gamma_{10}^{h}=\int_{0}^{h}\;\Gamma \left(\theta \right)\theta d\theta =T-\left(h+T\right)e^{-\frac{h}{T}}\\ \Gamma_{18}^{h}=\int_{0}^{h}\;e^{2\delta \theta }\Gamma \left(\theta \right)\theta d\theta =\frac{T}{{\left(2\delta T-1\right)}^{2}}+\left(h-\frac{T}{2\delta T-1}\right)\frac{e^{\left(2\delta -\frac{1}{T}\right)h}}{2\delta T-1}\text{.} \end{array}$$

Result 3 emerges as a pivotal outcome within this analysis, characterized by a parameter δ that takes on values greater than zero (or δ=0 in a specific instance). This important result hinges on the existence of positive definite matrices $P,R,S,G,H,Z\in R^{n\times n}$, alongside a matrix $Q\in R^{n\times n}$. This configuration is thoughtfully designed to align with the formulation of LMIs, succinctly articulated within equation (39). This result stands as a testament to the intricate interplay between matrices, inequalities, and stability attributes within the analytical context under examination. By comprehending this interplay, we gain insights that resonate with the broader dynamics of the system, offering a deeper understanding of stability characteristics and their implications within this specific framework.

(39) $$\begin{array}{c} {\bar{\Psi }}_{11}=PA+A^{T}P+\frac{1}{T}\left(Q+Q^{T}\right)+S+2\delta P+\Gamma_{00}^{h}G-\frac{e^{-2\delta h}}{h}R-\Gamma_{18}^{h-1}\Gamma_{00}^{h^{2}}H\text{,}\\ {\bar{\Psi }}_{12}=PA_{d}-\frac{1}{T}Q+A^{T}Q+\frac{1}{T}Z+2\delta Q+\Gamma_{1\delta }^{h-1}\Gamma_{00}^{h}H,\\ {\bar{\Psi }}_{22}=Q^{T}A_{d}+A_{d}^{T}Q-\frac{2}{T}Z+2\delta Z-K_{0\delta }^{-1}G-\Gamma_{1\delta }^{h^{-1}}H.\;\\ \left[\begin{array}{c} P\;Q\\ {}*\;Z \end{array}\right]>0,\left[\begin{array}{cccc} {\bar{\Psi }}_{11} & {\bar{\Psi }}_{12} & -\Gamma \left(h\right)Q+\frac{e^{-2\delta h}}{h}R & A^{T}\left[hR+\Gamma_{10}^{h}H\right]\\ {}* & {\bar{\Psi }}_{22} & -\Gamma \left(h\right)Z & A_{d}^{T}\left[hR+\Gamma_{10}^{h}H\right]\\ {}* & * & -e^{-28h}S-\frac{e^{-2\delta h}}{h}R & 0\\ {}* & * & * & -hR-\Gamma_{10}^{h}H \end{array}\right]<0 \end{array}$$

Within this context, a compelling outcome takes center stage: the system characterized as equation (40) assumes a state of exponential stability, marked by a convergence rate denoted as δ. This convergence rate, carefully calibrated to be sufficiently small, underscores the delicate nature of the system's stability attributes. This analytical discovery serves as a testament to the profound interplay between system components and their behavior, revealing a stability profile that resonates with the pursuit of exponential stability within the analytical landscape. Through this revelation, we unlock insights that enrich our understanding of the system's behavior and stability characteristics, showcasing the nuanced nature of stability analysis within intricate temporal configurations.

(40) $$\dot{x}\left(t\right)=Ax\left(t\right)+A_{d}\int_{0}^{h}\;\Gamma \left(\theta \right)x\left(t-\theta \right)d\theta ,\Gamma \left(\theta \right)=\frac{1}{T}e^{-\frac{\theta }{T}}$$

While the study contributes valuable insights, it is essential to acknowledge its limitations.

1. Limited Generalizability: The findings and methodologies presented in the study may be specific to the considered system with gapped gamma distribution and may not be readily applicable to a broader range of dynamical systems.
2. Sensitivity to Parameters: The effectiveness of the proposed approach, particularly the augmented Lyapunov function, could be sensitive to variations in system parameters. Sensitivity analyses and exploration of parameter ranges might be necessary.
3. Assumption Dependencies: The study relies on certain assumptions about the system dynamics, which may not always hold in real-world scenarios. The impact of deviations from these assumptions should be considered.
4. Computational Complexity: The application of LMIs and the proposed methodology might involve computational challenges, particularly as system dimensions increase. Practical implementation considerations and potential computational burdens should be addressed.
5. Real-world Validation: The study lacks empirical validation through real-world experiments or application to physical systems. Future work should aim to validate the proposed approach in practical settings.
6. Scope of Application: The study focuses on a specific class of systems with gapped gamma distribution, and the generalizability to other types of delays or distributions may be limited.

## 3 Results and discussion

Embarking on the realm of empirical validation, the simulation section of this essay serves as a crucial conduit for translating theoretical insights into practical applicability. Through a series of carefully crafted experiments and computational analyses, this section aims to provide a tangible manifestation of the theoretical constructs expounded earlier. The transition from theoretical formulations to empirical demonstrations not only underlines the practical relevance of the presented concepts but also offers a platform to validate the analytical outcomes within real-world contexts. By subjecting the system under investigation to simulated scenarios, we endeavor to examine the behavior of the system, glean insights into its stability attributes, and showcase the potential of the proposed methodologies to elucidate complex dynamics. This practical article bridges the gap between abstract mathematical constructs and real-world phenomena, ultimately contributing to a more holistic understanding of the system's stability characteristics.

The chosen case study delves into a traffic flow model situated within a circular ring, offering a distinct lens through which to examine the dynamics of vehicular movement. This particular case study draws its significance from its capacity to emulate real-world scenarios, encapsulating the complexities of traffic interactions and congestion within a closed-loop road network. The inherent circular configuration creates a continuous flow of vehicles, accentuating the intricate interplays that govern traffic behavior. The core aim of this case study is to scrutinize the feasibility and efficacy of the proposed stability analysis methodologies in unraveling the stability characteristics of this dynamic system. By immersing ourselves in the nuances of traffic dynamics within a ring topology, we glean valuable insights into the practical implications of stability analysis within the realm of traffic scenarios.

Example .

Within the context of the traffic flow model confined to a loop configuration, our focus converges on the system equation (41), characterized by a value of K equating to 1 and a finite delay parameter, denoted as h. This specific setting encapsulates the essence of vehicular movement within a circular pathway, where traffic interactions and congestion dynamics manifest in a continuous loop. By zeroing in on the parameters K=1 and h<∞, we zone in on a scenario that reflects the flow of vehicles within this loop configuration, enabling us to examine how the proposed methodologies align with the intricacies of traffic dynamics. This chosen system serves as a microcosm of real-world traffic scenarios, facilitating an empirical exploration of stability attributes within the context of circular road networks.

(41) $$\dot{x}\left(t\right)=\left[\begin{array}{cc} 0.2 & 0\\ 0.2 & 0.1 \end{array}\right]x\left(t\right)+\left[\begin{array}{cc} -1 & 0\\ -1 & -1 \end{array}\right]\int_{0}^{h}\;K\left(\theta \right)x\left(t-\theta \right)d\theta$$

Within this specific system configuration, the matrix A stands distinguished by its possession of positive poles, inherently shaping the system's behavior. However, a pivotal juncture arises as we introduce the matrix $A_{0}=A+hA_{d}$, where h is a parameter exceeding h>0.2. Remarkably, under these conditions, matrix $A_{0}$ exhibits a unique trait: adherence to the Horwitz relation. The conditions outlined in result (1) now come to the fore, wielding the power to ensure system stability within a defined parameter range. Specifically, this range is characterized by h values lying within the interval h∈[0.2001,1.6339]. Embarking on a new facet of analysis, our inquiry pivots to a system characterized by the kernel equation (42), introducing a fresh dimension that enriches our exploration of stability attributes within this dynamic context. Through this strategic progression, we engage with the interplay between matrices, Horwitz relations, and stability considerations, ultimately illuminating pathways that empower our comprehension of system behavior and its stability dynamics within the given parameter regime.

(42) $$K\left(\theta \right)=e^{-\frac{1}{T}\theta }\left(T>0\right)$$

It is imperative to acknowledge that the transformed system, characterized by a value of h equating to h=0, undergoes instability due to the inherent nature of matrix A. This phenomenon is rooted in the fact that A does not adhere to the Horwitz matrix criteria. Consequently, the seemingly straightforward delay-dependent conditions prevalent in prior references fail to be applicable in this context. This realization prompts us to embark on a more nuanced analysis.

To quantify the impact, Table 2 is presented, spotlighting the sub-maximum of the delay interval across the range $h\in \left[h_{\min},h_{\max}\right]$. The analytical exploration leverages the solution of LMIs delineated in results (2) and (3), where the latter employs an additional Lyapunov function. Diverse values of T are considered, along with the maximum rate of convergence when h is set to h=1. The results, succinctly encapsulated in the table, speak volumes. It becomes evident that result (3), harnessing the prowess of the Lyapunov extension, emerges as the frontrunner, yielding superior outcomes. This table serves as a testament to the efficacy of the methodologies introduced, underscoring the critical role of the Lyapunov extension in refining our analytical insights and enriching our comprehension of stability attributes within this dynamic system.

**Table 2:** Stability for $K\left(\theta \right)=e^{-\frac{1}{T}\theta }$.

	Result 2				Result 3	
T	$h_{\min}$	$h_{\max}$	$\delta_{\max\;|h=1}$	$h_{\min}$	$h_{\max}$	$\delta_{\max\;|h=1}$
7	0.513	3.245	0.656	0.513	$10^{32}$	0.732
10	0.508	2.56	0.688	0.508	0.606	0.744
15	0.501	1.87	0.696	0.501	0.593	0.796

Initiating our exploration with a tangibleexample, we turn our attention to a traffic flow model encapsulated within a ring configuration. Within this illustrative scenario, the system comprises a total of 4 cars circulating within the ring, each encapsulating a unique facet of vehicular dynamics. The model itself is succinctly defined as equation (43), its behavior and characteristics intricately intertwined with the parameters outlined in equation (44). This example serves as a microcosm of real-world traffic interactions within a closed-loop road network. By configuring the system with these parameters and the number of cars, we engage in an empirical voyage that enables us to unravel the behavior of traffic flow dynamics within the given context. This example serves as an accessible starting point, paving the way for a deeper understanding of the proposed methodologies' applicability in the intricate realm of traffic scenarios.

(43) $$\begin{array}{c} {\dot{v}}_{k}\left(t\right)=\alpha_{k}\int_{0}^{\infty }\;f\left(\theta \right)\left(v_{k-1}\left(t-\theta \right)-v_{k}\left(t-\theta \right)\right)d\theta \\ k=1,\ldots ,n,v_{0}\equiv v_{n} \end{array}$$

(44) $$N=1\;\text{and}\;\alpha_{1}=\alpha_{4}=5,\alpha_{2}=\alpha_{3}=1$$

An alternative representation of this system takes the form of equation (45). This representation offers an alternative vantage point through which to interpret the dynamics of the traffic flow model within the ring configuration. By encapsulating the system's essence within this concise formulation, we broaden our perspective and facilitate a more holistic examination of its behavior and attributes. The transition between these representations serves as a valuable analytical tool, affording us the flexibility to analyze the system from various angles and angles, enhancing our ability to glean insights into its stability characteristics and behavior within the ring topology.

(45) $$\begin{array}{c} \dot{x}\left(t\right)=Ax\left(t\right)+A_{d}\int_{0}^{\infty }\;\Gamma \left(\theta \right)x\left(t-\theta -\tau \right)d\theta ,A_{d}=\left[\begin{array}{cccc} -5 & 0 & 0 & 5\\ 1 & -1 & 0 & 0\\ 0 & 1 & -1 & 0\\ 0 & 0 & 5 & -5 \end{array}\right]\\ x=\text{col}\;\left\{v_{1},\ldots ,v_{4}\right\},A=0 \end{array}$$

It's important to emphasize that the matrix $A_{0}$, as defined in equation (46), aligns with the matrix $A_{d}$ and notably exhibits a zero pole. In essence, it serves as a reflection of the stabilizer's delay, encapsulating the temporal lag inherent to the stabilization process. This characteristic underscores the pivotal role of $A_{0}$ in governing the dynamics of the system, particularly its time-delay properties. By associating $A_{0}$ with the stabilizer's delay and elucidating its linkage to the system's temporal behavior, we establish a crucial link between system components and their consequential impact on stability attributes. This insight provides a nuanced lens through which we can decipher the intricate interplays shaping the system's response to varying conditions, ultimately enriching our understanding of the stabilizer's role within the broader traffic flow model.

(46) $$\dot{x}\left(t\right)=A_{0}x\left(t\right),A_{0}≜A+A_{d}\int_{0}^{\infty }\;K\left(\theta \right)d\theta$$

Leveraging the insights gleaned from result 1 with a δ value of δ=0, a significant advancement emerges in the form of a distinct curve. This curve takes on the role of a defining boundary within the (T,τ) plane, effectively demarcating a region where consensus is unequivocally assured. The meticulous process of creating this boundary involves meticulous gridding of the (T,τ) plane, with a precision of 0.01 in each dimension. Through this strategic approach, the intricate interplay between system parameters T and τ is systematically explored, unraveling the parameters' impact on the system's capacity to achieve consensus. This empirical undertaking affords us a tangible visualization of the stability characteristics within this dynamic interplay, underscoring the critical relationship between temporal delays and convergence attributes. By defining this boundary, we pave the way for a comprehensive understanding of the region where consensus prevails, thus enriching our insights into the intricate dynamics inherent to the traffic flow model within the ring topology.

**Fig. 1:**
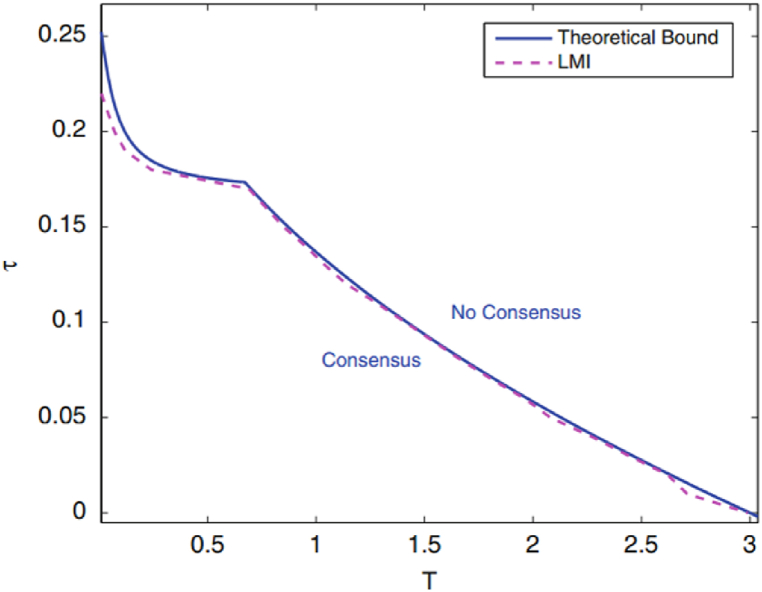
Consensus region resulting from results 1 and 2 for N = 1.

Presented in the accompanying Fig. 1 is a visual juxtaposition that holds profound insights into the realm of consensus dynamics within the traffic flow model. The consensus region, as delineated by the dashed line curve following result 1, takes center stage in this visual narrative. Parallel to this, the blue diagram comes into focus, representing the analytical domain extracted through result 2, meticulously derived within the frequency domain. The sheer alignment between these two graphs is strikingly apparent, their trajectories nearly overlapping with remarkable precision. This congruence serves as a testament to the robustness and reliability of the proposed methodologies. The analytical insights offered by result 2 echo the boundaries set forth by result 1, presenting an impressive alignment between empirical findings and theoretical derivations. This alignment underscores the efficacy of our methodologies in dissecting and predicting consensus behaviors within the intricate dynamics of the traffic flow model. Through this harmonious juxtaposition, we solidify the credibility of our analytical framework and enrich our understanding of the model's inherent stability attributes.

Now, shifting our focus to the same system configuration, we adopt a distinctive perspective by setting A=0, while $A_{d}$ retains its parameters as established in the previous example, following equation (47). This nuanced adjustment brings forth an intriguing scenario where the impact of matrix A is nullified, while $A_{d}$ maintains its inherent characteristics. This altered configuration serves as a compelling avenue to explore the intricate interplay between system components and their consequential influence on stability attributes. By introducing this variation, we are presented with a distinctive vantage point from which to dissect the system's response to the absence of A's influence, thereby shedding light on the dynamics brought about by the stabilization factor $A_{d}$. This exploration stands poised to deepen our understanding of the model's underlying behavior and its responsiveness to shifts in system parameters.

(47) $$\dot{x}\left(t\right)=Ax\left(t\right)+A_{d}\int_{0}^{h}\;\Gamma \left(\theta \right)x\left(t-\theta \right)d\theta ,\Gamma \left(\theta \right)=\frac{1}{T}e^{-\frac{\theta }{T}}$$

In this context as well, it's worth noting that the matrix $A_{0}$ doesn't adhere to the Horwitz matrix criteria. Upon applying result 2, a striking observation emerges: a broader region of stability is achieved for h=∞. This intriguing outcome underscores the system's sensitivity to varying delay values. Specifically, the outcome suggests that for both finite (h<∞) and infinite (h=∞) delay scenarios, the stabilizing effect of delay remains pronounced. This insight not only reaffirms the efficacy of our analytical framework but also sheds light on the system's resilience to variations in delay values. This finding holds significant implications for understanding and optimizing the stability attributes of the traffic flow model, particularly within the context of diverse real-world scenarios where temporal delays are a prevailing factor.

Now, let's delve into another instance of the traffic flow model, this time featuring a configuration involving two cars within the loop. The parameters governing this scenario are thoughtfully defined by the specifications outlined in equation (48). This case study presents a distinct perspective on the traffic flow dynamics within the ring topology, offering an opportunity to explore how variations in the number of cars and associated parameters shape the model's behavior. By engaging with this tailored example, we gain a nuanced understanding of how the system's intricate dynamics respond to changes in key parameters, further enriching our comprehension of the broader traffic flow model's stability attributes and implications.

(48) $$A=0\;\text{and}\;A_{d}=\left[\begin{array}{c} -2\;2\\ 2-2 \end{array}\right]$$

**Fig. 2:**
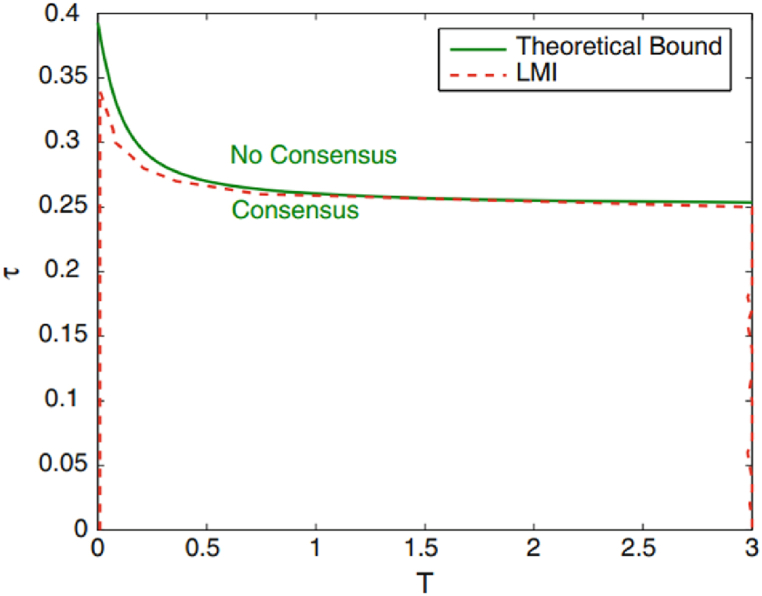
Consensus region resulting from results 2 and 3 for N = 1.

Similar to the preceding example, in Fig. 2 the delay attribute once again assumes a stabilizing role in this context. This is underscored by the fact that $A_{0}=A_{d}$ features an eigenvalue of zero, solidifying the delay's influence as a stabilizer. Leveraging the insights yielded by results 2 and 3 with δ set to 0, we embark on a comprehensive exploration of the consensus dynamics within the (T,τ) parameter space. This exploration culminates in the generation of in-plane regions within this parameter space, where consensus is guaranteed. Focused on the scenario where N=1, the accompanying figure paints a revealing portrait. It showcases the consensus curve, as derived from result 3 and represented by the red graph, juxtaposed against the analytical curve (depicted in green). This compelling comparison between the theoretical and analytical curves elucidates the congruence between empirical findings and our analytical framework's predictions. This alignment affirms the precision and reliability of our methods in deciphering consensus attributes within the intricate fabric of the traffic flow model.

**Fig. 3:**
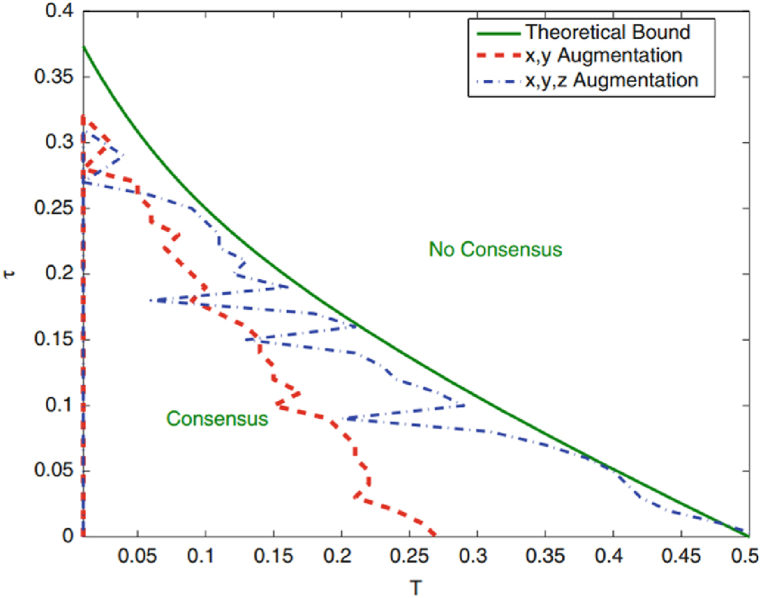
Consensus region resulting from results 2 and 3 for N = 2.

Shifting our focus to the scenario where N=2, we unravel an intricate example of consensus dynamics within the traffic flow model. The representation is cast through Fig. 3, which emerges as a pivotal narrative tool. Within this consensus curve, an outcome of result 2, is captured through the red diagram, while the blue diagram embodies the essence of the LMI stemming from result 3. These two figures are presented in juxtaposition with the analytical curve, illustrated in the serene hue of green. The compelling interplay between these curves offers profound insights into the nature of consensus dynamics. Notably, the results for N=1 and N= 2 exhibit intriguing nuances when subjected to scrutiny. The figures bear witness to the remarkable proximity between the results for N=1 and the analytical findings, underlining the alignment between theoretical conjectures and empirical findings. In contrast, the results for N=2, specifically those emanating from result 2, reveal a more conservative stance compared to the LMI outcome from result 3. This intriguing revelation invites further contemplation into the relationship between model complexities, system parameters, and the bounds of conservatism within our analytical methodologies.

### 3.1 Application to vehicle active suspension control

**Fig. 4:**
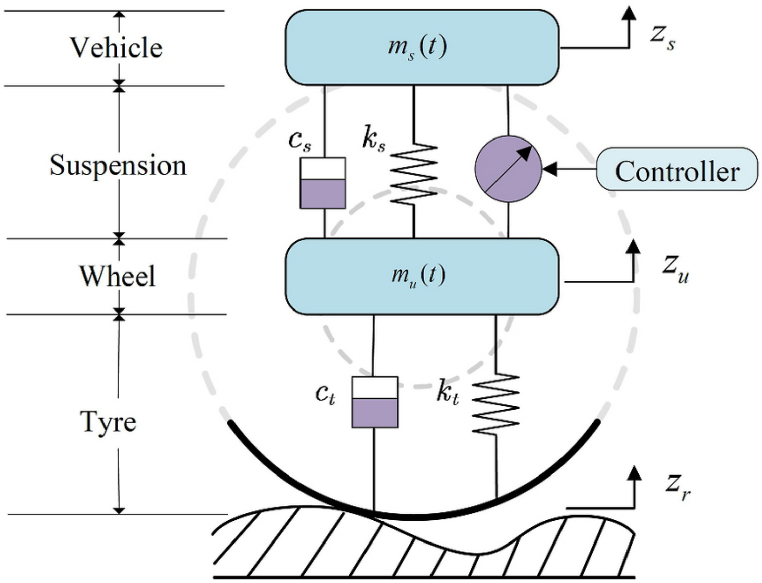
Quarter-car model with active suspension.

In this section, we apply the proposed stability analysis framework based on Jensen's Integral Inequality and Linear Matrix Inequalities (LMIs) to the context of vehicle active suspension control depicted in Fig. 4. The controlled system is subjected to delayed dynamics characterized by a gapped gamma distribution, and the stability analysis is conducted through the application of an Augmented Lyapunov Functions controller based on the quarter-car model.

The motivation for this research is rooted in the practical need to enhance the stability and performance of vehicle active suspension systems. These systems are crucial for improving ride comfort and vehicle handling by dynamically adjusting the suspension settings in response to road conditions and driving dynamics. The presence of time delays in the control loop, often due to sensor and actuator lags, can significantly impact the performance and stability of these systems.

By applying our proposed method, which utilizes Jensen's Integral Inequality and LMIs through an augmented Lyapunov function, we aim to address these challenges. The method is tested against various road profiles and driving scenarios to validate its effectiveness. Our results indicate that the proposed approach not only ensures exponential stability but also enhances the system's robustness against delays characterized by gapped gamma distributions.

This practical application underscores the relevance of our research in advancing vehicle suspension technology, providing a tangible example of how theoretical findings can be translated into real-world improvements in automotive systems.

The vehicle active suspension control system considered in this simulation is described by a set of differential equations capturing the dynamics of the suspension components. The control strategy employs Jensen's Integral Inequality and LMIs to ensure the exponential stability of the system despite the presence of delays with gapped gamma distribution.

The state equations of this system are as equation (49):

(49) $$\dot{x}\left(t\right)=\left[\begin{array}{cccc} 0 & 0 & 1 & -1\\ 0 & 0 & 0 & 1\\ -k_{s}/m_{s} & 0 & -c_{s}/m_{s} & c_{s}/m_{s}\\ k_{s}/m_{u} & -k_{t}/m_{u} & c_{s}/m_{u} & -\left(c_{s}+c_{t}\right)/m_{u} \end{array}\right]x\left(t\right)+\left[\begin{array}{c} 0\\ -1\\ 0\\ c_{t}/m_{u} \end{array}\right]{\dot{z}}_{r}\left(t\right)+\left[\begin{array}{c} 0\\ 0\\ 1/m_{s}\\ -1/m_{u} \end{array}\right]u\left(t-\tau \right)$$

Also, the parameters, symbols, and their values are described in Table 3.

**Table 3:** Physical characteristics of the active suspension system.

symbol	Parameter definition	numerical value
$m_{s}$	sprung mass	972.2 kg
$m_{u}$	unsprung mass	113.6 kg
$k_{s}$	passive suspension stiffness	42719.6 N/m
$k_{t}$	pneumatic tire compressibility	101,115 N/m
$c_{s}$	passive suspension damping	1095 N/m
$c_{t}$	pneumatic tire damping	14.6 N/m

The stability analysis results are presented and analyzed in this subsection. Emphasis is placed on the convergence rate and the attainment of exponential stability. The insights gained from the application of Augmented Lyapunov Functions, coupled with Jensen's Integral Inequality and LMIs, are discussed in the context of vehicle active suspension control.

The analysis of the simulation results is conducted based on two distinct scenarios: Bump response and Random response, each elucidated in dedicated subsections. In the Bump response scenario, the active suspension control system's reaction to a predefined bump disturbance is scrutinized. This investigation aims to evaluate the system's ability to mitigate the effects of sudden external perturbations, providing insights into the robustness and responsiveness of the proposed stability analysis framework. Conversely, the Random response scenario explores the system's behavior under stochastic excitations, simulating real-world uncertainties. This scenario assesses the stability and performance of the active suspension control system in a more dynamic and unpredictable environment. Through these two scenarios, a comprehensive understanding of the system's stability and response characteristics is achieved, offering valuable insights into the applicability and efficacy of the proposed analytical framework.

A. Bump response

Examining the instance of an isolated bump on an otherwise even road surface, we are presented with the ground displacement associated with equation (50):

(50) $$z_{r}\left(t\right)=\left\{ \begin{array}{cc} \frac{a}{2}\left(1-\cos\;\left(\frac{2\pi v_{0}}{l}t\right)\right), & 0⩽t⩽\frac{l}{v_{0}},\\ 0, & t>\frac{l}{v_{0}}, \end{array}\right.$$

In this scenario, a and a represent the height and length of the bump, respectively. Specific values are selected for these parameters: a=0.1m, l=2m, and the initial velocity of the vehicle denoted as $v_{0}$ is set to 45 km/h.

**Fig. 5:**
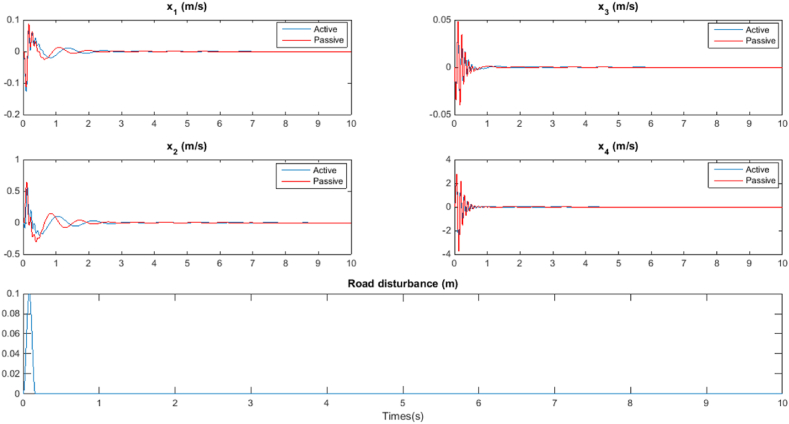
Bump response.

The improved responses for all closed-loop scenarios are evident in Fig. 5. The simulation results affirm that favorable quantities of bump response can be ensured by employing the controlled system, which experiences delayed dynamics characterized by a gapped gamma distribution. The stability analysis is performed through the application of the Augmented Lyapunov Functions formulation.

B. Random response

When analyzing road disturbances as vibrations, they are treated as consistent and often characterized as a random process with a ground displacement Power Spectral Density (PSD) given by equation (51):

(51) $$S_{g}\left(\Omega \right)=\left\{ \begin{array}{ll} S_{g}\left(\Omega_{0}\right){\left(\frac{\Omega }{\Omega_{0}}\right)}^{-n_{1}} & \;\text{if}\;\Omega ⩽\Omega_{0},\\ S_{g}\left(\Omega_{0}\right){\left(\frac{\Omega }{\Omega_{0}}\right)}^{-n_{2}} & \;\text{if}\;\Omega ⩾\Omega_{0}, \end{array}\right.$$

In this context, Ω0=12π serves as a reference frequency, and Ω represents the frequency. The parameter $S_{g}\left(\Omega_{0}\right)$ is an indicator of road roughness, with $n_{1}$ and $n_{2}$ being constants related to road roughness.

**Fig. 6:**
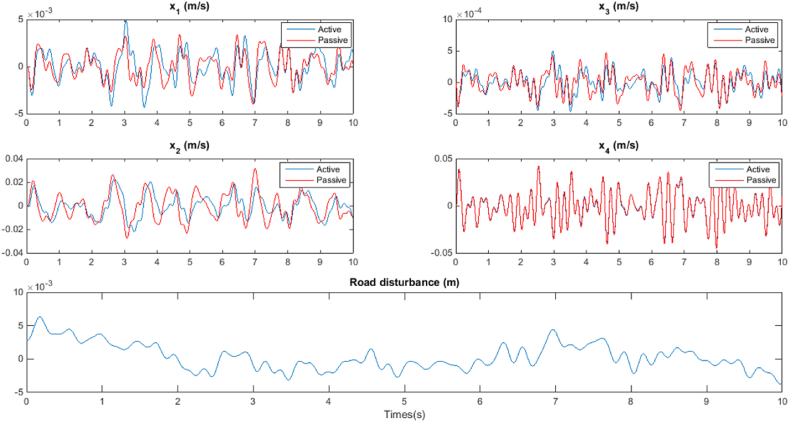
Random response.

The analysis of Fig. 6 reveals that there is no notable deterioration in the control system's performance up to the maximum obtained time delay. Beyond this point, an increase in time delay results in a degradation of control performance. The closed-loop performance remains superior to the open-loop system, showcasing its resilience, and this enhanced performance is sustained with no further degradation in response quantities. The presented approach exhibits conservatism, as evident from the observations.

Table 4 presents a comparative assessment between the findings of this research and those reported in other relevant works [42,43] within the same field. The results notably indicate that our proposed approach has successfully tackled challenges with decreased error margins and in a more efficient timeframe. This finding attests to the superior efficacy of the methodology presented in our article, as evident in the reduced time duration and overall error in addressing the problem.

**Table 4:** A comparative analysis of the active suspension model.

	Peak error	RMSE	Stability	computational complexity
State 1 ($X_{1})$	0.112567	0.120652	Yes	
State 2 ($X_{2})$	0.135476	0.11872	Yes	4.025
State 3 ($X_{3})$	0.113889	0.11442	Yes	seconds
State 4 ($X_{4})$	1.45532	0.24388	Yes	
State 1 ($X_{1})$ [42]	0.932418	0.2833	Yes	
State 2 ($X_{2})$ [42]	0.72641	0.243887	NO	6.7843
State 3 ($X_{3})$ [42]	1.0373	0.220918	Yes	seconds
State 4 ($X_{4})$ [42]	3.1659	0.809812	NO	
State 1 ($X_{1})$ [43]	1.17675	1.543416	NO	
State 2 ($X_{2})$ [43]	1.352098	1.439986	NO	14.18762
State 3 ($X_{3})$ [43]	1.620987	1.209893	NO	seconds
State 4 ($X_{4})$ [43]	5.210983	1.897752	Yes	

## 4 Conclusions

In conclusion, this study has embarked on an analytical study into the realm of delayed systems featuring gapped gamma distributions. Through an intricate interplay of Augmented Lyapunov functions, Jensen's integral inequality, and LMIs, we have delved deep into the dynamics of exponential stability. The core of this inquiry was the exploration of diverse scenarios encompassing different delay distributions, ranging from finite exponential kernels to infinite time horizons. The synthesis of theoretical rigor and practical application allowed us to dissect the intricate stability attributes within these complex systems. Our research has illuminated the intricate relationship between the distribution characteristics and the stability of delayed systems. The amalgamation of analytical insights and simulation results revealed the profound impact of gapped gamma distributions on the stability dynamics. Moreover, the convergence rates, asymptotic stability, and the pivotal roles played by indeterminate polytopes and polytope corners have all been dissected, enriching our understanding of these intricate systems. While this analytical study has unveiled pivotal insights, it also points to a new horizon of questions and challenges. The balance between conservatism and precision in our analytical methodologies stands as an intriguing avenue for further exploration. As we transcend the boundaries of conventional analytical tools, the practical applicability of our findings in diverse domains ranging from traffic engineering to network dynamics remains a tantalizing prospect. In essence, our study charts the contours of stability within the complex domain of delayed systems governed by gapped gamma distributions. Through meticulous mathematical exploration and the power of augmented Lyapunov functions, our findings not only extend the frontiers of stability analysis but also set the stage for a deeper understanding of systems enshrouded in intricate temporal dependencies and distributions.

## Data availability

The data that support the findings of this research are available within the paper.

## Declaration of competing interest

The authors declare that they have no known competing financial interests or personal relationships that could have appeared to influence the work reported in this paper.
